# A powerful molecular synergy between mutant Nucleophosmin and Flt3-ITD drives acute myeloid leukemia in mice

**DOI:** 10.1038/leu.2013.77

**Published:** 2013-04-19

**Authors:** A Mupo, L Celani, O Dovey, J L Cooper, C Grove, R Rad, P Sportoletti, B Falini, A Bradley, G S Vassiliou

**Affiliations:** 1 The Wellcome Trust Sanger Institute, Wellcome Trust Genome Campus, Hinxton, Cambridge, UK; 2 Hematology and Clinical Immunology Section, Department of Clinical and Experimental Medicine, University of Perugia, Perugia, Italy

Acute myeloid leukemia (AML) is the commonest myeloid malignancy, yet there has been little therapeutic progress for this disease in decades, and only 25–30% of patients survive long term.^1^ This reflects its pathogenetic complexity and the fact that the molecular basis of its largest cytogenetic subgroup, AML with a normal karyotype (AML-NK), was obscure until recently. Recent advances in DNA sequencing have revealed that AML-NK is molecularly heterogeneous with >30 genes recurrently targeted by somatic mutations in this disease.^2^ What is also evident is that each individual case of AML-NK appears to harbor only a small number of coding driver mutations, often as few as three and usually no more than five.^2, 3^ Furthermore, it is manifest that the precise combination of driver mutations in the genome of each AML impacts on its salient features, including responsiveness to treatments and prognosis.^3^

These observations provide a sound starting point for systematic mechanistic studies to understand the pathogenesis and improve the treatment of AML-NK. Carefully designed mouse models are the gold standard in the study of normal and malignant hemopoiesis, and are already instructing our understanding of AML-NK.^4, 5^ Here, we report that the two most commonly co-occurring somatic mutations in AML, namely Nucleophosmin (*NPM1*) exon 12 mutations (*NPM1c*) and internal tandem duplications of *FLT3* (*FLT3-ITD*), cooperate explosively to induce AML in knock-in mice. In revealing this striking molecular synergy, our work offers a basis for the frequent co-occurrence of these two mutations and provides a valuable model for in-depth studies of the pathogenesis and treatment of this large subgroup of AML.

NPM1 is a nucleolar phosphoprotein involved in many cellular processes. For many of its roles, it relies on its ability to shuttle between the nucleolus, nucleus and cytoplasm using subcellular localization signals.^6^ This ability is impaired in 30% of AMLs as a result of *NPM1c* mutations, which disrupt the nucleolar localization signal of NPM1 and generate a nuclear export signal in its place.^7^ Mutant NPM1 is known to bind to and alter the subcellular distribution of several proteins, including HEXIM1, p19Arf and nuclear factor-κB;^8^ however, the relevance of these interactions to AML is unclear. *FLT3-ITD* mutations occur in 20–25% of AML^9^ and result in ligand-independent receptor dimerization and constitutive FLT3 signaling,^10^ and are associated with an increased risk of relapse. Moreover, patients with low or absent levels of wild-type (WT) FLT3, consistent with loss-of-heterozygosity (LOH) for this locus, have a particularly poor outcome.^9^

Recently, we described a conditional knock-in mouse model of NPM1c mutations and demonstrated that one-third of mice developed delayed-onset AML, suggesting a requirement for cooperating mutations. We went on to show that insertional mutagenesis with transposons led rapidly to AML in 80% of *Npm1c* mice, in association with specific recurrent mutations including activating insertions in *Csf2* and *Flt3*.^4^ *Flt3-ITD* homozygous mutant mice exhibit enhanced proliferation and survival properties in hemopoietic progenitors and develop a late-onset disease akin to chronic myelomonocytic leukemia.^11^

To study the combined effects of *NPM1c* with *FLT3-ITD* we crossed conditional *Npm1*^*flox−cA/+*^ with constitutive *Flt3*^*ITD/+*^ to generate *Npm1*^*flox−cA/+*^*; Flt3*^*ITD/+*^ double heterozygous mice, then crossed into *Mx1-Cre* transgenic mice to induce recombination of *Npm1*^*flox−cA*^ in hematopoietic stem cells.^4^ The *Mx1-Cre* allele requires induction by interferon, usually achieved by intraperitoneal injection of polyinosinic-polycytidylic acid(pIpC). However, we observed universal and rapid emergence of AML (myeloid leukemia with maturation) in uninjected *Npm1*^*flox−cA*^*;FLT3*^*ITD/+*^*;Mx1-Cre+* mice (hereafter referred to as ‘*Npm1c/Flt3-ITD* mice'). *Mx1-Cre* is known to ‘leak' in 2–4% of hemopoietic stem/progenitor cells,^12^ and this was sufficient to rapidly generate AML from double mutant cells signifying a striking cooperativity between *Npm1c* and *Flt3-ITD*. The presence of the cytoplasmic NPM1 was confirmed on protein blots (Figure 1a).

All *Npm1c/Flt3-ITD* mice developed AML and became moribund aged 31–68 days (median 49 days; *n*=29). By contrast, no case of AML was observed in *Npm1*^*flox−cA*^*;Mx1-Cre+* mice (hereafter referred to as ‘*Npm1c* mice' *n*=30, of which 15 received pIpC aged 6–8 weeks), *FLT3*^*ITD/+*^ mice (hereafter referred to as ‘*Flt3-ITD* mice' *n*=34) or WT mice (WT, *n*=29) aged to at least 8 months (Figure 1b). Weekly blood counts from 19 mice with each genotype showed a progressive increase in blood leukocyte counts in *Npm1c/Flt3-ITD* mice, to more than 25-fold that of age-matched control littermates, whereas the hemoglobin and platelet counts were significantly reduced (Figure 1c).

Interestingly, *Npm1c/Flt3-ITD* siblings/littermates often progressed to AML at different rates or developed more/less aggressive disease. To explain this observation we hypothesized that, as seen in human AML, LOH for *Flt3-ITD* may be responsible. We found evidence for significant spontaneous loss of the WT *Flt3* allele in blood samples from *Npm1c/Flt3-ITD* mice and a tendency for higher blood leukocyte counts (Figure 1d) when LOH was present. LOH was also seen in bone marrow and spleen but not tail DNA, in keeping with somatic loss of the WT allele in leukemic cells (Figure 1d). At the time mice became sick with AML, LOH was detected in 12 of 15 spleen samples tested.

Flow cytometric analysis of blood samples demonstrated, in *Npm1c/Flt3-ITD* mice, a population of blasts/immature cells with low side scatter (SSC) and CD45^dim^ (Figure 2a) and a large number of single Mac1+ precursors (Figure 2b). In addition, we also observed an increased number of mature myeloid (Gr1+/Mac1+) cells in *Npm1c/Flt3-ITD* mice, indicating that any maturation block was incomplete (Figure 2b). The relative numbers of circulating B (B220+) and T (CD3+) lymphocytes were reduced (data not shown). To assay their self-renewal potential, bone marrow cells from *Npm1c* (*n*=4), *Flt3-ITD* (*n*=4), *WT* (*n*=4) and *Npm1c/Flt3-ITD* (*n*=4) were studied in serial replating assays. *Npm1c/Flt3-ITD* cells gave rise to significantly more colonies at first and subsequent platings than any other genotype (Figure 2c), demonstrating a significantly increased self-renewal potential.

Blood smears from sick mice confirmed the presence of blasts, and histological sections demonstrated widespread infiltration of solid organs by abnormal myeloid cells (Supplementary Figure S1). Cells infiltrating the bone marrow and spleen were Gr1+/Mac1+ or Gr1−/Mac1+, and there were increased numbers of Mac1+/cKit+ cells compared with other genotypes (Supplementary Figure S2). Compared with single mutant and WT mice, sick *Npm1c/Flt3-ITD* mice had marked splenomegaly (0.95±0.27 g vs 0.13±0.02 g; *P*<0.0001) and hepatomegaly (2.33±0.26 g vs 1.6±0.17 g, *P*<0.0001) at the time of death. *Npm1c/Flt3-ITD* leukemias were transplantable into both syngeneic and NOD SCIDγ mice demonstrating their true neoplastic nature (data not shown).

AML is a molecularly and clinically heterogeneous disease and recent studies have revealed that this heterogeneity is derived, to a large extent, from the specific combinations of somatic driver mutations present in individual cases. Here, we show that the combination of *Npm1c* and *Flt3-ITD*, the two most commonly co-occurring AML mutations, is rapidly and universally leukemogenic in knock-in mice. These findings are particularly striking in light of the fact that, in isolation, both *Npm1c*^4^ and *Flt3-ITD*^11^ mutations have relatively subtle effects on mouse hemopoiesis and lead to leukemia or a myeloproliferative disorder only after prolonged latencies and in a minority of mice.

What is most remarkable about our findings is the very short latency of AML in *Npm1c/Flt3-ITD* mice, which suggests either: (i) that the two mutations are sufficient to promote AML in this strain of mice (C57BL6/N) or (ii) that additional mutations are acquired very rapidly in the pool of cells susceptible to leukemic transformation. The later possibility is supported by the fact that at least one type of somatic mutation, namely LOH for *Flt3-ITD*, was frequently observed in our mouse AMLs over this short time span. We recently reported that *Npm1c* can generate AML in collaboration with, amongst others, activating insertions of the *GrOnc* transposon in intron 9 of mouse *Flt3*. These insertions led to aberrant expression of a *Flt3* messenger RNA predicted to code for an amino-terminal truncated version of Flt3^4^ which, like Flt3-ITD, was thought to be constitutively active. Most of these murine AMLs harbored additional transposon insertions thought to be important in leukemogenesis. Thus, at this stage it appears more likely that additional mutations may be required for leukemogenesis in our *Npm1c/Flt3-ITD* mice, but this cannot be stated unequivocally.

In interesting contrast to our present work, a recent report demonstrated that the combination of *Flt3-ITD* with *NUP98-HOXD13* in mice led to AML after a much longer latency (median 95 days),^14^ despite the fact that, unlike *Npm1c*, *NUP98-HOXD13* alone leads to a highly penetrant myelodysplastic syndrome with a high risk of leukemic transformation. This relative delay is particularly intriguing as *NUP98-HOXD13* can promote leukemic transformation in association with simple overexpression of WT *FLT3*.^15^ By contrast, in two large transposon-mediated insertional mutagenesis screens, one published^4^ and one ongoing, we never observed transposon insertions leading to simple *Flt3* overexpression amongst >100 mouse *Npm1c* +ve AMLs.

Notwithstanding the above, our observations emphasize the remarkable complementarity between *Npm1c* and *Flt3-ITD*. In the context of a stochastic model for AML pathogenesis,^2^ this potent molecular synergy goes some way toward explaining why *NPM1c* and *FLT3-ITD* co-occur so frequently and make the model described here a valuable tool for the study of the pathogenesis and treatment of one of the largest molecularly defined subgroups of AML.

## Figures and Tables

**Figure 1:**
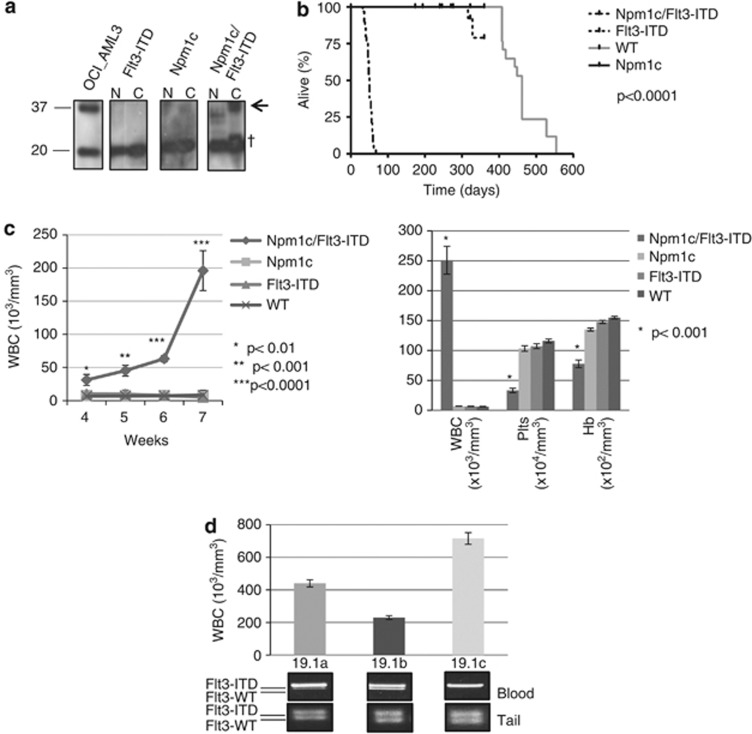
*Npm1c* and *Flt3-ITD* collaborate to drive rapid-onset leukemogenesis with frequent occurrence of *Flt3* LOH. (**a**) Npm1 mutant protein (arrow) accumulates in the cytoplasm of spleen cells collected from 3-week-old *Npm1c/Flt3-ITD*, but not *Npm1c* or *Flt3-ITD* single-mutant mice. (**b**) Kaplan-Mayer survival plots showing the rapid demise of *Npm1c/Flt3-ITD* mice compared with all other genotypes. (**c**) Serial blood counts highlight a consistent explosive increase in blood leukocytes counts (WBC) between 4 and 7 weeks in *Npm1c/Flt3-ITD* mice (left) and the markedly abnormal WBC, platelet count (Plts) and hemoglobin concentration (Hb) of sick leukemic *Npm1c/Flt3-ITD* mice compared with age-matched control mice. (**d**) Loss of the *Flt3* WT allele in blood DNA from *Npm1c/Flt3-ITD* AMLs is demonstrated as loss of intensity of the *Flt3*-WT PCR band. By contrast, constitutional tail DNA shows no LOH. In these three littermates (19.1a–c), the extent of Flt3-LOH associates with the degree of leukocytosis (N=nuclear lysate, C=cytoplasmic lysate, OCI-AML3 lysate as positive control, † nonspecific band).

**Figure 2:**
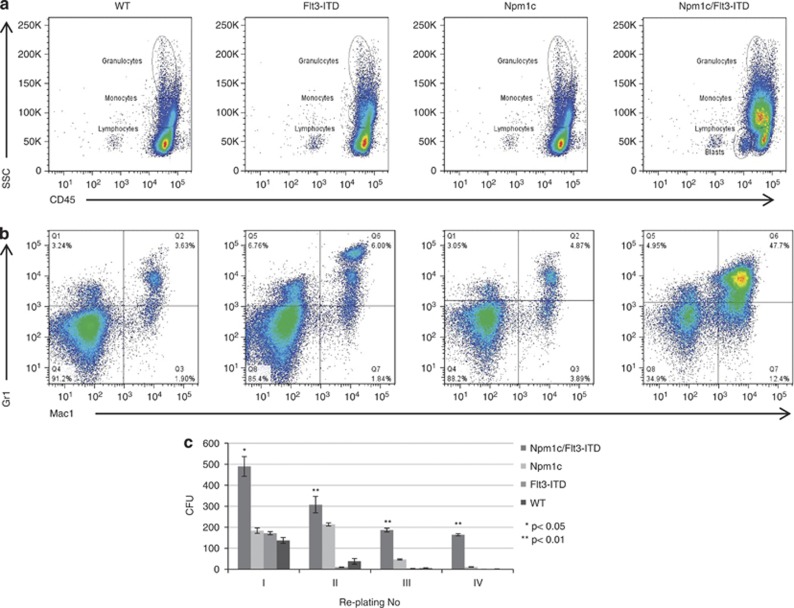
Expansion of circulating myeloid cells in *Npm1c/Flt3-ITD* mice culminating to aggressive AML. (**a**) Representative flow cytometric analysis of peripheral blood from 7-week-old WT, *Npm1c*, *Flt3-ITD* and *Npm1c/Flt3-ITD* mice shows the presence of a low SSC; CD45^dim^ population of immature/blast cells in double mutant mice and (**b**) an increase in both mature granulocytic (Gr1^+^/Mac1^+^) and monocytic (Gr1^−/lo^/Mac1^+^) populations. (**c**) Colony-forming assays of BM cells derived from WT and mutant mice showing a markedly increased replating ability of *Npm1c/Flt3-ITD* cells compared with other genotypes, indicative of an increased self-renewal potential. As we described before, a lesser increase in replating ability is observed with *Npm1c* cells.
