# Supplementary figures and images for: A powerful molecular synergy between mutant Nucleophosmin and Flt3-ITD drives acute myeloid leukemia in mice
Source: Leukemia. 2013 Apr 19;27(9):1917–20. doi: 10.1038/leu.2013.77 (PMC3768110; doi:10.1038/leu.2013.77)

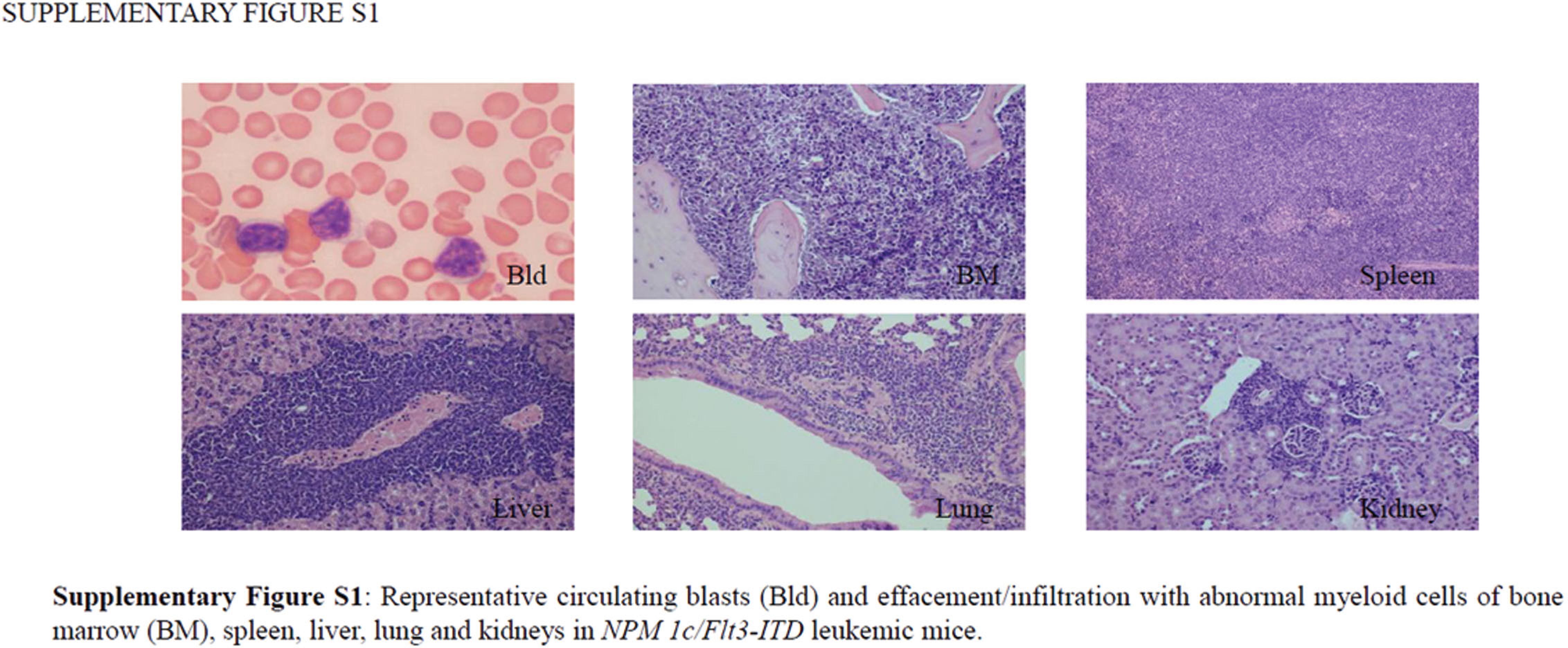

Supplement: Supplementary Figure S1 [file leu201377x1.tif]

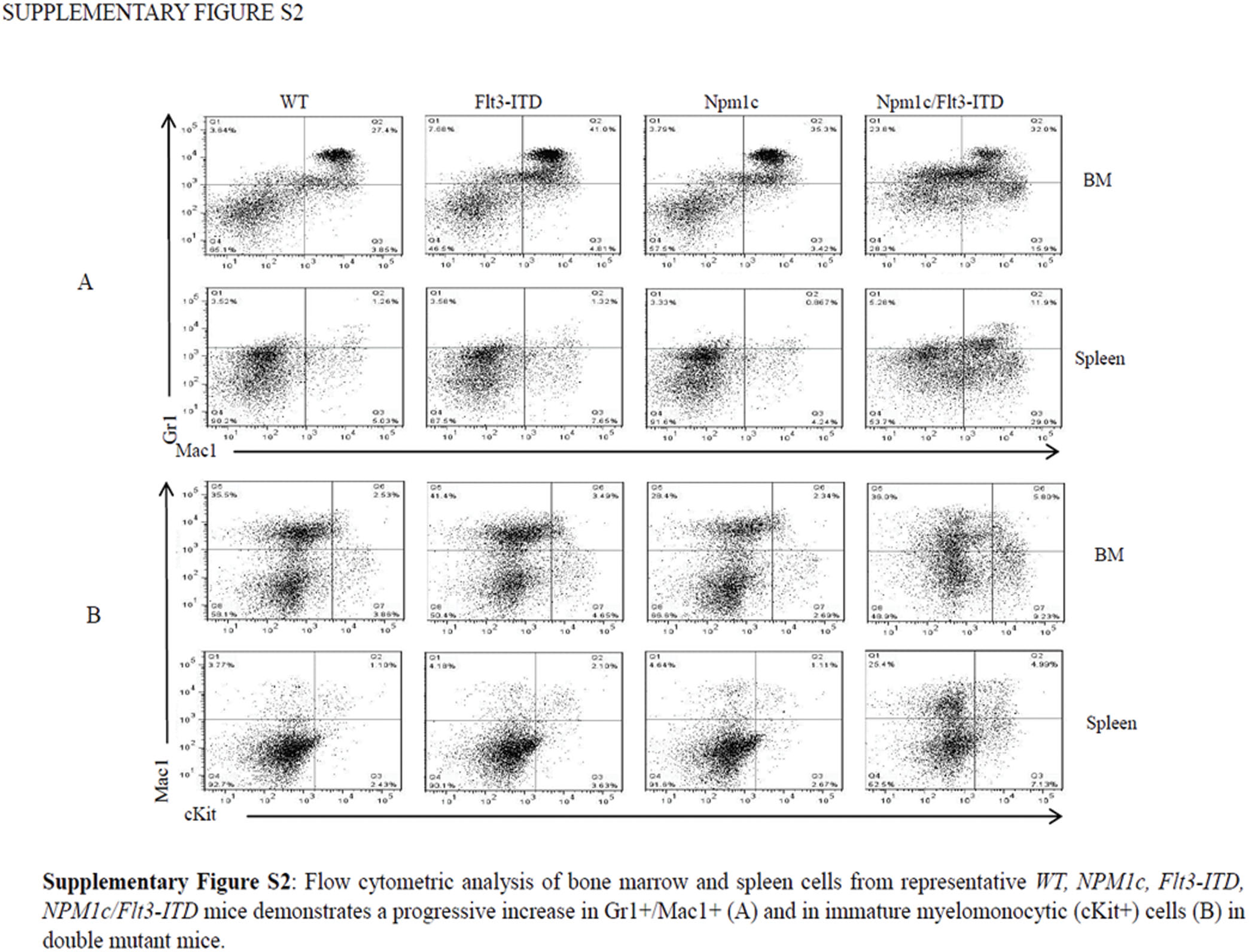

Supplement: Supplementary Figure S2 [file leu201377x2.tif]
